# Supplementary material for: Lessons learned from the adaptation of the Reflective Functioning Questionnaire (RFQ) for Dutch people with mild to borderline intellectual disabilities
Source: PLoS One. 2023 Jun 27;18(6):e0287751. doi: 10.1371/journal.pone.0287751 (PMC10298756; doi:10.1371/journal.pone.0287751)
Supplement: S1 File — (PDF) [file pone.0287751.s001.pdf]

THE REFLECTIVE FUNCTIONING QUESTIONNAIRE FOR PEOPLE WITH MILD TO BORDERLINE INTELLECTUAL DISABILITIES

You can ask your caregiver or someone else to complete this list with you.

Name:

Date of birth:

Gender:

You will see statements.
These statements are about you.
Please indicate whether you agree with this statement.
Do you agree or disagree?
Give the answer that suits you best.

Question	Do you agree or disagree? (Tick 1 option per statement)						
	Strongly disagree	Disagree	Little disagree	Not agree, not disagree	Little agree	Agree	Strongly agree
1. I don't understand what people think.							
2. I don't always know why I do something.							
3. When I am angry I say things without knowing why.							
4. When I am angry I say things I don't want to say.							
5. When I don't feel safe, I sometimes do things that other people don't like.							
6. Sometimes I do things and I don't know why.							
7. Strong feelings make it hard to think.							
8. It is easy for me to know what other people are thinking and feeling.							
9. I find it hard to understand other people's feelings.							
10. The thoughts and feelings of other people confuse me.							
**Total score subscale Self** 0							
**Total score subscale Other** 0							

Extra explanation per question

Below you see extra explanations for each question. Also, you can split the answer for each question in two. First, you can decide if you disagree, not agree/ not disagree or agree. Then decide to what extent you agree or disagree (strong to little).

Question	Extra explanation
1. I don't understand what people think.	Do you understand what people think? Then you disagree with this question. Do you find it very difficult to understand what other people think? Then you agree with this question.
2. I don't always know why I do something.	Do you have a thought about why you do things? About the reason why you do something? Then you disagree with this question. Don't you have a thought about why you do things? Then you agree with this question.
3. When I am angry I say things without knowing why.	When you are angry, do you sometimes say things without a reason? Then you agree with this question. Do you say things with a reason when you are angry? Then you disagree with this question.
4. When I am angry I say things I don't want to say.	When you are angry, do you sometimes say things you would rather not have said? Then you agree with this question. When you never say things you shouldn't have said when you are angry? Then you disagree with this question.
5. When I don't feel safe, I sometimes do things that other people don't like.	By not feeling safe, we mean that you don't feel comfortable in the situati-on. It is about being in a situation where you don't want to be anymore. And when you feel this, do you sometimes do things that others don't like? Then you agree with this question. Don't you do things that others don't like, when you don't feel safe? Then you disagree with this question.
6. Sometimes I do things and I don't know why.	Do you ever do things without having a clear reason / without any thoughts behind it? Then you agree with this question. Don't you do things without having a clear reason/ without any thoughts behind it? Then you disagree with this question.
7. Strong feelings make it hard to think.	Strong feelings are very strong emotions. For example if you are very angry or very sad. How you feel can affect how you think. Do you find it harder to think if you have strong feelings? Then you agree with this question. Don't you find it harder to think when you have strong feelings? Then you disagree with this question.
8. It is easy for me to know what other people are thinking and feeling.	Can you easily tell what someone is thinking or feeling? Then you agree with this question. Can't you easily tell what someone is thinking or feeling? Then you disagree with this question.
9. I find it hard to understand other people's feelings.	Do you find it difficult to understand other people's feelings? Then you agree with this question. Do you find it easy to understand other people's feelings? Then you disagree with this question.
10. The thoughts and feelings of other people confuse me.	Do you ever get confused by the thoughts and feelings of others? Then you agree with the question. Do you never get confused by the thoughts and feelings of others? Then you disagree with this question.

Total scores subscales

Subscale	Discription of the subscales
Self	Subscale focused on the self and one's own feelings and thoughts. High scores on this subscale indicate the uncertainty to understand one's own complex mental states.
Other	Subscale focused on the self in relation to the feelings and thoughts of others. High scores on this subscale indicate the uncertainty to understand others complex mental states.

The original Reflective Functioning Questionnaire (RFQ) contains items 1 to 7. Items 8 to 10 are additional items added to the RFQ for people with mild to borderline intellectual disabilities. With the additional items there are two subscales RFQ Self and RFQ Other. For more information about the original RFQ see: Fonagy et al. (2016)

© Fonagy et al. (2016) – RFQ (London, United Kingdom)
© S.D.M. Derks, A.M. Willemen & P.S. Sterkenburg (2022) – RFQ for people with MBID (Amsterdam, The Netherlands)
25-04-2022
