# Supplementary material for: Lessons learned from the adaptation of the Reflective Functioning Questionnaire (RFQ) for Dutch people with mild to borderline intellectual disabilities
Source: PLoS One. 2023 Jun 27;18(6):e0287751. doi: 10.1371/journal.pone.0287751 (PMC10298756; doi:10.1371/journal.pone.0287751)
Supplement: Attachment — Submitted filename: Response to the reviewers.docx [file pone.0287751.s002.docx]

Date	First submission number

1-7-2022	PONE-D-22-05515

**Response to the reviewers**.

We thank the reviewers for reviewing our manuscript ‘*Psychometric properties of an adapted, Dutch version of the Reflective Functioning Questionnaire (RFQ) for people with mild to borderline intellectual disabilities’.* We very much appreciate the feedback we received. We are also grateful for the opportunity to revise our manuscript and submit a new version of the manuscript for consideration as a new submission. We believe that the manuscript is really improved.

Below we respond in more detail to the feedback from the reviewers.

Reviewer 1

We thank the reviewer 1 for reviewing our manuscript. We were pleased to read that the reviewer indicates our article as interesting and find the theoretical premises and the aim of the study clear. We will respond to the comments below.

2.1) Comment: An important limitation, as also recognized by the same authors, is the use of a self-report scale to assess the ToM ability. Generally, I suggest using performance measures the mentalising abilities, specifically when evaluated people with mild to borderline intellectual disabilities. Self-reported functioning reflects the perception that a person has about his/her performance of tasks; whereas performance-based measures are more objective and assess the ability to perform a specific task correctly or incorrectly. The negative significant correlations between the Frith-Happé animations and the original RFQ certainty subscale is the proof that self-report and performance measure, even if evaluate the same ability, aren't congruent.

There are several mentalising performance measures in literature; why did the authors select the RFQ?

Response: The aim of this study was first of all to investigate if it is possible to measure mentalizing with a self-report instrument in adults with mild to borderline intellectual disabilities. In addition, we also want to use this research to serve practice, and thus develop a short, easy to use instrument that can also provide professionals with insight into mentalizing of our target group. Moreover, reporting and thinking of their own abilities through self-report measures stimulate the autonomy, self-determination, and independence of our target group^[1]^.

The RFQ as instrument was chosen because it is a relatively short instrument that appear psychometrically sound. Moreover, its items refer to reflection on daily life, having the opportunity to measure reflection on both the self and others. Research into measuring mentalizing in our target audience is still in its infancy. The Frith-Happé animations test in our study measures Theory of Mind that is related to mentalizing but mainly focused on the other and external cues. Therefore, we also acknowledged in our discussion that: ‘*Studies should further investigate the relationship between the RFQ and other, experimental measures of mentalisation that still need to be adapted to adults with MBIDs.*’ (page 24).

2.2) Comment: The Authors didn't use a comprehension test to assess whether the participants had a good level of comprehension. It would have been interesting to have the WAIS indices including the Verbal Comprehension.

Response: We thank the reviewer for this suggestion. In our adaptation process, we checked for comprehension of the RFQ. No major problems emerged from this check. In addition, we added to our limitation section the recommendation: ‘*However, the level of comprehension was not specifically examined in our study. Future studies may need to examine associations between IQ and the level of comprehension tested with for example Wechsler Adult Intelligence Scale (WAIS) [46] and the usability of the RFQ*.’ (page 24).

2.3) Comment: It is unclear why the authors choose AQ as the convergent measure; this test does not measure an ability or competence, but the presence of autistic traits. Generally, the choice of convergent and divergent validity measures is not clear.

Response: In our new manuscript, we thoroughly revised our introduction and the hypotheses for convergent and divergent validity measures. Testing convergent validity, the definition as given in Abma et al. (2011)^[2]^ was leading, namely: ‘.. verifies whether the scores of the instrument under study “make sense” in relation to the scores of other, related instruments.’ (p. 1). First we indicate in our Introduction that ‘*Furthermore, the presence of autistic traits may impede development and suggest limitations on mentalising skills or their manifestation, especially in mentalising related to others’ thoughts and feelings’* (page 5). Second, based on an earlier psychometric properties study of the RFQ also including autism in their research (Morandotti et al., 2018^[3]^), we can state that autistic traits, measured here with the AQ10, can be used to investigate convergent validity.

In addition, we added to our Introduction: ‘*Stronger correlations were expected with the other-oriented items compared to the self-oriented items, because the other-oriented focus is also represented in autistic traits (e.g., greater challenge with other people's thoughts and feelings) and perspective taking.*’ (page 7).

For divergent validity, we added to our Introduction: ‘*Divergent validity was investigated by associations between the RFQ-8 and perspective taking, emotion recognition and Theory of Mind, expecting non-significant correlations as the RFQ-8 is primarily internal and self-oriented while the other concepts reflect the external or internal and other-oriented dimensions of mentalising*’ (page 7).

Reviewer 2

Next, we greatly thank reviewer 2 for the kind words. We are delighted to read that the reviewer also indicates our study as interesting and well written, and also thinks that our study could make a relevant addition given its specific target group and extension of the content of the questionnaires.

We moreover thank the reviewer for the critical thoughts on ‘the applicability and validity of the CFA approach taken to establish the factorial structure of the test, and as a result also about the actual interpretability and validity of this suggested structure and the two resulting scores for certainty and uncertainty for both the original and extended RFQ.’ We agree with the comments on the questionable CFA model. Again, carefully looking at recent studies that indeed criticizes the approach of scoring the RFQ^[4],^^[5]^, we revised our manuscript and analyses thoroughly. The comments of the reviewer were very helpful.

We invite the reviewer to re-read our manuscript, but in summary we investigate ‘*the proposed one-factor structure of the RFQ-8 as suggested by Müller et al. [18] and Woźniak-Prus et al. [19]. Second, in co-creation, we selected some additional items from the 54-item version of the RFQ that focus on the self in relation to the thoughts and feelings of others. We investigated the factor-structure including the additional items*.’ (page 7, Introduction).

‘*Factor structure was determined first by conducting a confirmatory factor analysis (CFA) in AMOS (version 26) for the unidimensional model with the RFQ-8 adapted for people with MBID*.’ (page 14, Materials and Methods). ‘*Second, exploratory factor analysis (EFA) was conducted to examine the factor structure of the RFQ including the additional items. EFA was performed with Principal Components analysis. Scree plot and parallel analysis [33] were used to determine the number of factors. When applicable, the factor structure found in the EFA was evaluated in a CFA*.’ (page 15, Materials and Methods).

We finally concluded that: ‘*The results of the current study showed adequate model fit for the unidimensional RFQ-8, as proposed Müller et al. [18] and Woźniak-Prus et al. [19]. In addition, an adequate model fit was found for a two-factor structure with the subscales ‘RFQ Self’, representing reflection on the self and one’s own feelings and thoughts, and ‘RFQ Other’, representing reflections on the self in relation to the feelings and thoughts of others.*’ (page 21, Discussion).

Again, we thank the reviewers for their time and efforts to improve the paper as the use of the RFQ in this target group is new and offers opportunities for research and acquiring knowledge about mentalisation abilities in this population that can be expanded through more research and knowledge from practical use.

1. United Nations. (2006). *Convention on the Rights of Persons with Disabilities (CRPD).*
2. Abma, I. L., Rovers, M., & van der Wees, P. J. (2016). Appraising convergent validity of patient-reported outcome measures in systematic reviews: constructing hypotheses and interpreting outcomes. *BMC Research Notes, 9*(1), 1-5.
3. Morandotti, N., Brondino, N., Merelli, A., Boldrini, A., De Vidovich, G. Z., Ricciardo, S., ... & Luyten, P. (2018). The Italian version of the Reflective Functioning Questionnaire: Validity data for adults and its association with severity of borderline personality disorder*. PLoS One, 13*(11), e0206433.
4. Müller, S., Wendt, L. P., Spitzer, C., Masuhr, O., Back, S. N., & Zimmermann, J. (2021). A critical evaluation of the Reflective Functioning Questionnaire (RFQ). *Journal of Personality Assessment*, 1-15.
5. Woźniak-Prus, M., Gambin, M., Cudo, A., & Sharp, C. (2021). Investigation of the Factor Structure of the Reflective Functioning Questionnaire (RFQ-8): One or Two Dimensions?. *Journal of Personality Assessment*, 1-11.
